# Methotrexate and actinomycin D chemotherapy in a patient with porphyria: a case report

**DOI:** 10.1186/s13256-015-0790-6

**Published:** 2016-01-18

**Authors:** Yukiko Mikami, Tomonori Nagai, Yousuke Gomi, Yasushi Takai, Masahiro Saito, Kazunori Baba, Hiroyuki Seki

**Affiliations:** Department of Obstetrics and Gynecology, Saitama Medical Center, Saitama Medical University, 1981 Kamoda, Kawagoe-shi, Saitama 350-8550 Japan

**Keywords:** Porphyria, Gestational trophoblastic disease, Methotrexate, Actinomycin D

## Abstract

**Background:**

Despite their broadly recommended use as chemotherapeutic agents, the porphyrogenicity of methotrexate and actinomycin D have not been confirmed. Accordingly, it is not known whether these agents are safe for use in patients with porphyria.

**Case presentation:**

In this report, we present a case of an invasive mole with lung metastasis in a 49-year-old Japanese woman who had previously been diagnosed with acute intermittent porphyria at 27 years of age but had no recent history of acute intermittent porphyria attacks. Her serum human chorionic gonadotropin level was elevated 1 month after hysterectomy, and she was referred to our center for chemotherapy. After she received 100 mg of methotrexate, drug eruptions were observed starting on day 3 and grew progressively worse. Erythema and mucosal erosion spread throughout her body, whereupon she was administered prednisolone. In addition, our patient experienced febrile neutropenia and required granulocyte colony- stimulating factor treatment. No changes in our patient’s urinary coproporphyrin or uroporphyrin levels were detected during this entire episode. Methotrexate was replaced by actinomycin D (0.5 mg/body intravenously on days 1–5 every 2 weeks). After five uneventful cycles of actinomycin D, our patient achieved and maintained a normal serum human chorionic gonadotropin level for 3 years.

**Conclusions:**

Methotrexate and actinomycin D did not induce acute porphyric attacks in this patient with acute intermittent porphyria; however, severe adverse effects were noted with methotrexate. Although further investigation is required, our data suggest that these agents are nonporphyrinogenic and can therefore be used to treat patients with comorbid porphyria.

## Background

In 2002, the International Federation of Gynecology and Obstetrics (FIGO) 2000 staging and risk factor scoring system, which recommends chemotherapy regimens for gestational neoplasia, were published by the FIGO Oncology Committee [1]. Among the recommendations, methotrexate (MTX) and actinomycin D (ACT-D) were the most commonly suggested chemotherapeutic drugs in various regimens. The folic acid antagonist MTX is most frequently used to treat gestational trophoblastic disease (GTD) [2], as well as leukemia and other cancers such as breast cancer and urothelial carcinoma. In addition, different doses of MTX can be used to treat rheumatoid arthritis and psoriasis vulgaris. However, MTX has been known to induce various toxicities, including interstitial pneumonia, hepatopathy, bone marrow suppression, bone destruction, stomatitis, and gastric ulcer. In addition to GTD treatment, ACT-D is used to treat Wilms’ tumor, Ewing sarcoma, and rhabdomyosarcoma; however, this agent has been reported to induce hepatic vein obstruction.

Porphyrias comprise a collection of seven disorders that result from genetic defects in heme biosynthesis. The clinical management priorities for patients with porphyria include the prevention of acute attacks and avoidance of skin lesions. Many factors have been identified to induce acute attacks, including porphyrinogenic drug use, fasting, smoking, alcohol consumption, and infection, as well as emotional and physical stress [3]. Accordingly, specific precautions must be taken when treating porphyric patients with malignant diseases in order to minimize the risk of an acute porphyric attack and to ensure optimal therapeutic effects against the malignancy. However, the porphyrinogenic or nonporphyrinogenic statuses of many chemotherapeutic drugs, including MTX and ACT-D, have not yet been established since the association of each chemotherapeutic drug and acute intermittent porphyria (AIP) is very rare. Therefore, it is essential that such data be accumulated and reported.

The present report details our experience with the treatment of an invasive mole with lung metastasis in a Japanese patient with AIP. Although a severe drug eruption as well as febrile neutropenia occurred in response to MTX treatment, our patient successfully completed chemotherapy without experiencing a porphyric crisis.

## Case presentation

A 49-year-old Japanese woman (gravida 4, para 3) was diagnosed with AIP at the age of 27. Our patient had repeatedly visited the hospital for stomachaches beforehand; however, approximately 2 years of consultations passed before she was accurately diagnosed. Afterwards, she avoided elements known to precipitate acute attacks, and had not experienced an acute porphyria attack since she was 33 years of age. In addition, she began hypertension treatment with amlodipine besylate at the age of 40 years, and her last pregnancy had spontaneously aborted 4 years prior to the present event. In July 2011, this patient visited a local private clinic for atypical vaginal bleeding and was referred to a general hospital because of suspected spontaneous abortion. She was subsequently diagnosed with a acute intermittent porphyria hydatidiform mole via dilatation and curettage (D&C).

**Fig. 1:**
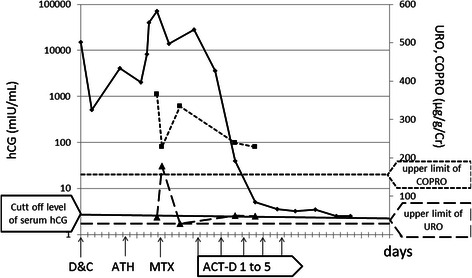
Changes in serum human chorionic gonadotropin (*hCG*) levels, urinary 8-carboxyl porphyrin (*URO*) and urinary 4-carboxyl porphyrin (*COPRO*) during treatment. *ACT-D* actinomycin D, *ATH* abdominal total hysterectomy, *D&C* dilatation and curettage, *MTX* methotrexate

One month after D&C, our patient presented with an elevated serum human chorionic gonadotropin (hCG) level (Fig. 1). As fertility preservation was deemed impossible, she underwent an abdominal total hysterectomy. Pathological study of the resected specimen revealed an invasive mole, but no metastasis was observed. However, our patient’s serum hCG level was again elevated 1 month after hysterectomy, and she was referred to Saitama Medical Center for chemotherapy to address expected AIP-associated complications. An X-ray computed tomography scan of her chest, performed at our institution, revealed a lung metastasis. The FIGO 2000 staging and risk factor scoring system indicated a stage III:41 gestational trophoblastic neoplasia (GTN) (Tables 1 and 2), and our patient was accordingly diagnosed with a low-risk GTN. Her treatment strategy included intramuscular MTX administration at a dose of 20 mg/day for 5 days every 2 weeks.

**Table 1:** FIGO 2000 risk factor scoring system for gestational trophoblastic neoplasia

Prognostic factors	Score			
	0	1	2	4
Age (years)	<40	≥40		
Antecedent pregnancy	Mole	Abortion	Term	
Interval from index pregnancy (months)	<4	4–7	7–13	>13
Pretreatment serum hCG (IU/mL)	<103	103–104	104–105	>105
Largest tumor size (including the uterus) (cm)	<3	3−5	≥5	
Metastatic site	Lung	Spleen, kidney	Gastrointestinal tract	Liver, brain
Number of metastases		1−4	5−8	>8
Previous failed chemotherapy			Single drug	≥2 drugs

Total score: ≤6, low risk; >7, high risk

*FIGO* International Federation of Gynecology and Obstetrics, *hCG* human chorionic gonadotropin, *IU* international unit

**Table 2:** FIGO 2000 staging for gestational trophoblastic neoplasia (GTN)

FIGO staging	
Stage I	Disease confined to the uterus
Stage II	GTN extends outside of the uterus, but is limited to genital structures (adnexa, vagina, and broad ligament)
Stage III	GTN extends to the lungs, with or without known genital tract involvement
Stage IV	Metastasis to other organ sites

*FIGO* International Federation of Gynecology and Obstetrics

**Fig. 2:**
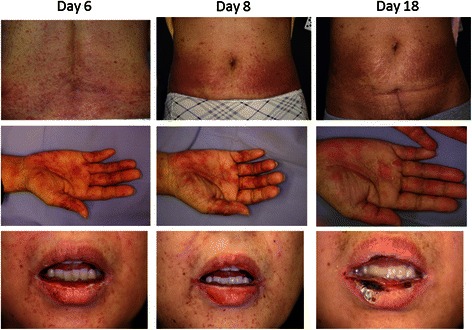
Progress of eruption. On day 6, erythema with infiltration appeared on the trunk (*upper panel*); blisters containing bloody serum developed on our patient’s hands (*middle panel*), and erosive lesions and a furred tongue were observed in her mouth (*lower panel*). On day 8, the erythema became brown (*upper panel*); bullous lesions were observed on her hands (*middle panel*), and mucosal erosion of her mouth continued (*lower panel*). On day 18, the erythema improved, leaving only pigmentation (*upper panel*); the bullous rash was epithelialized with scaling (*middle panel*), and some erosive lesions became blood-filled blisters (*lower panel*)

**Fig. 3:**
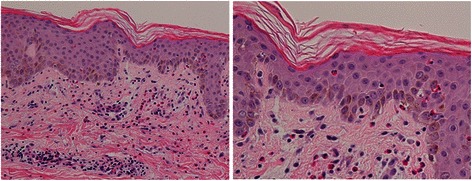
Abdominal skin biopsy. Microabscesses formed under the stratum corneum, with neutrophilic and eosinophilic invasion, and neutrophilic exocytosis was observed in the epidermis (hematoxylin and eosin staining, ×20 and ×40 magnification)

MTX chemotherapy was initiated after our patient provided informed consent, which included an understanding that MTX had not been implicated as either porphyrinogenic or nonporphyrinogenic. Our patient experienced small eruptions on the hands and epigastric region on day 3 of chemotherapy. However, a dermatologist did not interpret these eruptions as drug-related, and thus treatment was continued. On day 5, the eruptions expanded over her whole body and worsened, with ulcers developing on the oral mucosa (Fig. 2). Our patient was unable to eat or drink owing to the severe pain caused by mucosal erosion; moreover, she complained of painful micturition because of a sore on her vulva. A skin biopsy revealed drug eruption instead of a porphyric skin lesion. In particular, microabscesses had formed under the stratum corneum, with neutrophilic and eosinophilic invasion, and neutrophilic exocytosis was observed in the epidermis. Liquefaction was also observed between the epidermis and dermis, and eosinophilic, neutrophilic, and lymphocytic invasion from the layer between the epidermis and dermis to the superficial layer of the dermis was noted (Fig. 3). No changes in our patient’s urinary coproporphyrin or uroporphyrin levels were observed despite the appearance of skin lesions (Fig. 1). She received intravenous prednisolone at 60 mg/day beginning on day 5 to treat the drug eruption.

On day 11, our patient experienced febrile neutropenia (absolute neutrophil count [ANC] on day 8 was 1,380/μL but decreased to 12/μL on day 11) and was treated subcutaneously with 75 μg/day of granulocyte-colony stimulating factor (G-CSF) for 3 days beginning on day 11. However, as she was unable to recover from neutropenia (ANC remained at 12/μL on day 13), additional subcutaneous G-CSF at 150 μg/day was administered for 2 days beginning on day 13, and her neutropenia finally resolved on day 15 (ANC, 1598/μL). During that time, her skin lesions and stomatitis also began to improve, and the prednisolone dose was reduced to 40 mg/day. As she was able to eat on day 17, oral prednisolone administration at 20 mg/day was started on day 20 and tapered on day 24. She was discharged on day 25.

Although our patient’s serum hCG level decreased with MTX treatment, it became elevated during treatment interruption. Chemotherapy with ACT-D was therefore considered. After providing informed consent to ACT-D treatment and acknowledging that ACT-D was not implicated as either porphyrinogenic or nonporphyrinogenic, our patient received intravenous ACT-D at a dose of 1.5 mg/day on days 1–5 of every 2-week period, starting on day 70 of her clinical course. As shown in Fig. 1, her serum hCG level effectively decreased, and the lung metastasis disappeared without any porphyric attacks. After five cycles of ACT-D, her serum hCG level stabilized at 3.2 mIU/mL. Although other chemotherapeutic drugs were considered, a negative serum hCG level was achieved and maintained for the following 3 years.

## Discussion

Porphyrias represent a collection of seven disorders caused by inherent or acquired defects in genes related to heme biosynthesis. The accumulation of intermediate products such as porphobilinogen in the liver or skin induces porphyric symptoms. Partial deficiency of any of the seven enzymes in heme biosynthesis pathway results in characteristic clinical and biochemical features (Table 3), which are used to classify porphyrias into dermatological, neuropsychiatric, and mixed forms. Porphyria cutanea tarda (PCT) is the most common form, with characteristic predominant cutaneous manifestations such as photosensitivity. Neuropsychiatric (acute) porphyria is seen in AIP and plumboporphyria, and has various manifestations, including acute attacks. Such acute attacks are the most concerning manifestation of porphyria and are known to be induced by certain drugs, infection, alcohol consumption, smoking, fasting, and stress.

**Table 3:** Porphyria classification

Type of porphyria	Enzyme inheritance	Inheritance	Clinical manifestation			Urine concentration
			Skin	Neuropsychiatric		
Plumboporphyria	Porphobilinogen synthase	AR, 9q34	-	++		ALA COPRO-gen III
Acute intermittent porphyria	Hydroxymethylbilane synthase	AD, 11q23	-	++	Abdominal pain, neuropathy, mental changes, and tachycardia	PBG σ-ALA
Congenital erythropoietic	Uroporphyrinogen III synthase	AR, 10q26	+	-	Skin lesion and hepatopathy in early childhood	UPG I COPRO-gen I
Porphyria cutanea tarda	Uroporphyrinogen decarboxylase	AD, 1q34	++	-	Dark urine, occurring after middle age	UPG
Hereditary coproporphyria	Coproporphyrinogen oxidase	AD, 9	+	+	Abdominal pain and peripheral, neuropathy	PBG>ALA COPRO-gen III
Variegate porphyria	Protoporphyrinogen oxidase	1q14	+	+		PBG>ALA COPRO-gen III
Erythropoietic protoporphyria	Ferrochelatase	18q21, 3	+	-	Skin lesion and hepatopathy in early childhood	Not increased (PP in erythrocyte)

*AR* autosomal recessive, *ALA* aminolevulinic acid, *COPRO-gen* coproporphyrinogen, *AD* autosomal dominant, *PBG* porphobilinogen, *UPG* uroporphyrinogen, *PP* protoporphyrin

To date, many porphyrinogenic drugs have been identified (Table 4) [4, 5]. Safe treatment of concomitant diseases with nonporphyrinogenic drugs is the most important aspect of preventing acute attacks. However, some porphyrinogenic chemotherapeutic drugs without alternative options might be required for malignant diseases. A list of drugs known to be porphyrinogenic or nonporphyrinogenic according to animal studies and case reports is shown in Table 5 [6]. However, contradicting reports exist for a number of chemotherapeutic agents, including MTX.

**Table 4:** Contraindicated medications in porphyria

Antimigraine agents: ergot preparation
Antipsychotic drugs: ethchlorvynol, glutethimide, hydroxyzine
Cardiocirculatory drugs: hydralazine, lidocaine, methyldopa, spironolactone
Hormonal agents: danazol, progesterone
Analgesic drug: diclofenac
Anesthetic agent: barbiturate sedative
Antiseizure drugs: barbituric acid, carbamazepine, phenytoin, sodium valproate
Antibiotic: chloramphenicol, clindamycin, erythromycin, rifampicin, ketoconazole
Muscle relaxants: carisoprodol, orphenadrine
Respiratory drugs: clemastine, dimenhydrinate

**Table 5:** Carcinostatic medications and porphyria

Porphyrinogenic (chick studies):
Cyclophosphamide, azathioprine, 5-fluorouracil, busulfan, procarbazine, hexamethylmelamine
Porphyrinogenic (case reports):
Cyclophosphamide, busulfan, methotrexate
Nonporphyrinogenic (chick studies):
Dacarbazine, chlorambucil, melphalan
Nonporphyrinogenic (case reports):
Mitomycin C, cyclophosphamide, mitoxantrone, 5-fluorouracil, cytosine arabinoside, doxorubicin, 6-thioguanine, vincristine, procarbazine, methotrexate, actinomycin D, cisplatin

Several case studies have reported an association between PCT and MTX therapy [7–9]. Most porphyria-related drugs are thought to induce cytochrome P450 activity and thus increase the demand for hepatic heme biosynthesis, leading to increased porphyrin formation. However, as hepatic MTX metabolism does not involve cytochrome P450, the association between MTX and PCT cannot be explained by this mechanism. Additionally, PCT lesions were reported to disappear despite continued MTX therapy in at least one study [10]. Furthermore, experimental studies of chick embryos, which are believed to reflect the human susceptibility to drug-induced porphyria exacerbation, found no effects of MTX on porphyrin metabolism [10]. In our case, MTX induced severe cutaneous lesions that were histopathologically diagnosed as a drug eruption rather than porphyria. Drug eruption is a common adverse effect of various types of nonchemotherapeutic drugs, and it was highly unlikely that there was any relationship between cutaneous symptoms and porphyria in our patient because no changes in her urinary coproporphyrin or uroporphyrin levels were observed throughout her episode. Thus, MTX appeared to be nonporphyrinogenic in our case.

In contrast, ACT-D has been reported to be safe in AIP patients [11]. No case reports have indicated an association between this agent and porphyric attacks, and ACT-D was safely used in the case presented herein. Although more evidence from experimental studies and case reports is needed for a definitive conclusion, we believe that ACT-D is a potentially nonporphyrinogenic drug.

In cases not involving potentially porphyrinogenic chemotherapeutic agents, stresses such as sepsis from neutropenia and long fasting related to gastrointestinal toxicity or pain [6] could also induce acute attacks. Hematologic growth factors, including G-CSF, have been reported to be safe for porphyria patients [12], and this was true in our experience. Our results thus indicate that G-CSF can help to prevent acute attacks resulting from sepsis. Among commonly prescribed antiemetics, the 5-hydroxytryptamine receptor antagonist ondansetron and glucocorticoid are also considered safe for patients with porphyria [4, 5]. Additionally, dexamethasone can be used safely, but no evidence is available regarding aprepitant. Although alcohol consumption can induce a porphyric attack, there are no reports on the safety of taxane therapy in patients with porphyria.

## Conclusions

In conclusion, given the rare nature of porphyrias, knowledge regarding the safe use of chemotherapeutic agents in these patients will require evidence from additional case reports. We believe that our case contributes to this knowledge and supports the use of MTX and ACT-D as potentially nonporphyrinogenic chemotherapeutic agents.

## Patient’s perspective

I write the following to provide support for the case report written about my treatment. I have no medical knowledge or background so I can only write from my own perspective and experience.

I was diagnosed with acute intermittent porphyria (AIP) at 27 years of age. Prior to that, I had been treated in ambulatory centers and hospitals many times for abdominal pain, but the clinicians could not diagnose the cause of this pain. Although I experienced very severe pain, sometimes this was doubted and thought to be caused by a mental disorder.

After I was diagnosed with AIP, the doctors told me that many factors had been identified to induce acute attacks, and I avoided these factors. I did not experience any more abdominal pain attacks beginning at 33 years of age; however, I felt uneasy about receiving chemotherapy.

The doctors at Saitama Medical Center told me that methotrexate (MTX) was most commonly used to treat my disease (invasive mole with lung metastasis) but had not been implicated as either porphyrinogenic or nonporphyrinogenic, and therefore, they carefully treated me with MTX.

On day 3 of the first chemotherapy course, I noticed small eruptions on my hands and stomach, and reported this to the doctors in charge. Next, the dermatology specialist visited me for an examination; he judged my skin lesions to be mild drug-caused eruptions and allowed the treatment to continue. On day 5, the eruptions expanded over my whole body, and ulcers developed on my oral mucosa. I could not eat or drink anything, not even my own saliva. Each day, I discharged saliva onto paper because I could not swallow it. For me, this period when I could not drink and had a fever was the hardest. Fortunately, however, I did not experience abdominal pain similar to that in an AIP attack.

Two weeks after the initiation of chemotherapy, my skin symptoms improved and I was gradually able to eat a meal. My doctors said I would need to be treated with another chemotherapeutic drug because my serum hCG levels were elevated, but I wanted to go home at once instead. Therefore, I was discharged from the hospital after I became able to eat adequately.

My husband and I listened to an explanation about another anticancer agent from a doctor in the outpatient department 1 week later. She told us that the next medicine, actinomycin D, had also not been evaluated for use in patients with AIP. However, we understood the need for chemotherapy, and we agreed to its use.

I was hospitalized again and received the new chemotherapy regimen. In contrast to my fear, it did not cause any side effects. I continued to receive this therapy, being hospitalized and discharged again. My husband and I were very happy to learn that my serum hCG levels had decreased and the lung metastasis had disappeared. After five rounds of chemotherapy with actinomycin D, my serum hCG level plateaued at 3.2 mIU/mL. My doctors told me about another chemotherapy, and I was very disappointed. However, because I had been able to receive the second chemotherapy with few side effects, I convinced myself that I might survive the next chemotherapy.

While the doctors prepared a new chemotherapeutic drug, my serum hCG value fell below the cutoff value. The doctor told me that I would usually need to receive three additional courses of chemotherapy after the serum hCG level fell below the cutoff level, but my serum hCG level remained under the cutoff level 1 month after stopping the second chemotherapy. I had a long conversation with my doctor, and we decided to finish the chemotherapy at that time.

I began to work at an office 1 year after I finished this treatment. I am currently living well.

I felt uneasy about using medicines for which the safety in patients with AIP was unknown, so I will be very happy if my experience will help other patients with porphyria.

## Consent

Written informed consent was obtained from the patient for publication of this case report and any accompanying images. A copy of the written consent is available for review by the Editor-in-Chief of this journal.

## Abbreviations

ACT-D: actinomycin D
AIP: acute intermittent porphyria
ANC: absolute neutrophil count
D&C: dilatation and curettage
G-CSF: granulocyte-colony stimulating factor
GTD: gestational trophoblastic disease
GTN: gestational trophoblastic neoplasia
hCG: human chorionic gonadotropin
MTX: methotrexate
PCT: Porphyria cutanea tarda
